# Excerpta

**Published:** 1880-10

**Authors:** 


### EXCERPTA.

About the year 1858 attention was called to the subject of the appointment of dentists in the army and navy, and the writer who was at that time the editor of the *New York Dental Journal* took an active interest in the matter; but, although the subject was more or less agitated by the dental press throughout the country at intervals thereafter, it was not until August of the year 1861 that the American Dental Convention (at the session held in New Haven, Conn.) appointed a committee of five, consisting of Drs. W. H. Atkinson, Cleaveland, O.; G. H. Perine, New York; B. W. Franklin, New York; J. D. White, Philadelphia. and I. J. Wetherbee, Boston, to inquire into the advisability of the course. They conferred with Surgeon-General William A. Hammond, and the matter was very favorably received by him. But no positive action was taken until 1868, when a bill authorizing the appointment of dentists in the army and navy was drafted and presented before both houses of Congress by Senator Hamlin, of Maine, which was referred to the Committee on Military and Naval affairs, by whom, however, no decided steps were taken. A second bill was, during the Forty-Second Congress, laid before the House by the Hon. De Witt Townsend, advocating dental appointments at the United States military and naval academies, and, as before, the subject was referred to the committee, who failed to bestow upon it the attention so important a matter deserved. A dentist has, it is true, been appointed at the Annapolis Academy, with the rank of assistant surgeon, but beyond this nothing has been done in the direction referred to. It strikes us as not a little singular that a movement of such importance has of late received so little notice from the members of our specialty. To those who have given the subject consideration, the necessity of appointments of the character we refer to must be apparent. Sound teeth are among the physical requirements of soldiers and sailors, and certainly no physician or specialist will deny that attention to the preservation of these organs, does much towards preserving the health of those in our country's service, and that the evident lack of interest displayed by the government is highly reprehensible. For some years past the establishment of dental chairs in the State medical colleges and the treatment of dentistry as a specialty of medicine have been more or less agitated, and strongly advocated by a large number of the leading members of the profession, and it is doubtful whether any stronger argument can be advanced in favor of such a movement than that contained in this article. A union of dentistry with medicine would be a decided step gained in favor of the appointments herein suggested, in making which no additional expense would be incurred by the government. Doubtless much of the opposition which advocates of the cause have had to contend with has arisen from the fact that few, if any, of our army and navy surgeons possess a knowledge of dentistry, and that the appointment of physicians practicing our specialty, would necessitate a new order of things in this particular direction. At the military stations of the far West, and on board naval training-ships, the services of a dentist are often required, and much suffering is at times experienced from the lack of proper treatment of disease of the oral cavity. This fact in itself should prove a sufficient incentive to a vigorous movement on the part of not only those practicing our specialty, but physicians also. There is no excuse for the indifference displayed by the government in a matter bearing so directly upon the sanitary condition of its servants. There is little doubt but that those members of our specialty constituting State dental associations could, by a joint effort, enlist the attention of a sufficient number of our national representatives to insure the successful accomplishment of this very necessary measure.—*Cosmos.*

### THOMAS ON HEARING THROUGH THE TEETH AND CRANIAL BONES.

Dr. Thomas proposes the term "osteophone" for all appliances—including the audiphone and dentaphone—intended to aid hearing by conveying articulate sounds through the medium of the cranial bones. His researches lead him to the following amongst other conclusions: The audiphone is much better adapted for use at a distance than the dentaphone, the latter being only suited to transmit sounds emitted near its mouthpiece. Although these instruments are of great value in a considerable portion of cases, they supply, the author considers, a very small fraction of normal hearing—much *less* than a *hundredth part.* It is important that this should be taken into account, for a large number of partially deaf persons suffer such disappointment at their failure to hear in full that they undervalue or altogether disregard a positive gain of many times their usual hearing.

The very small fraction of normal hearing gained is, the author thinks, of priceless value in many cases of those who hear practically nothing without these instruments. In regard to deaf-mutes, the audiphone is worthless unless they possess the faculty of hearing their own voices without the instrument. The author has constructed an audiphone which can be kept in position without the use of the hand. The best material for diaphragms he finds to be Fuller's board (or press board) treated with shellac varnish. A simple rod of hard wood, one end of which is placed on the upper teeth of the speaker, the other on those of the listener, or on his head, acts as a powerful osteophone, and will transmit the vocal vibrations in great volume to the ears of the deaf person.—*The London Medical Record.*

### THE DE LESSEPS CANAL IN ITS RELATION TO HYGIENE.

The title of a paper before us by G. Halsted Boyland, M. A., M. D., etc. (reprint from the *Independent Practitioner*, Baltimore), will scarcely fail to attract the deserved attention of all sanitarians who would watch the results of drainage on a grand scale, through one of the most malarious regions in the world. Dr. Boyland, being in close relation with the eminent author of this great enterprise, has made excellent use of his opportunity to place the sanitary relations of the subject before the public under the light of the best literature, and, in anticipation, in comparison with the results of similar works, although on a much less gigantic scale in other places. He shows a clear appreciation of the physical conditions to be contended with, and although somewhat enthusiastic in his advocacy of this particular place and p'an, and admiration for its author, his attention is not by any means diverted from the particu'ar branch o: the subject which he has taken up, or in the least disposed to underrate its difficulties and dangers. On the contrary, he presents them as they are, and with all t'eir disadvantages still thinks them less than any change of place would involve, and would, therefore, measure his efforts to surmount them by the same scale.

It affords us especial satisfaction to be made the medium of a second paper from Dr. Boyland, in continuation of the subject, printed on other pages of this number, showing the lively appreciation of M. De Lesseps and his associates in the enterprise, of timely and adequate measures for the protection of the health of the operatives in the work.—*The Sanitarian.*

Treatment of Anal Fissure.—Dr. Hamon informs us, in *Le Praticien*, that, instead of employing forcible dilatation, he applies to the fissure, with a camel-hair brush, a solution consisting of one part of chloroform to two parts of alcohol. Two or three applications, at intervals of two or three days, usually suffice to effect a cure. The first application is very painful, but each subsequent one becomes less so.—*Canada Medical and Surgical Journal.*

Treatment of Perspiration of the Feet.—Dr. Ortega (*La Prat.*) advocates the use of a solution of chloral in this affection (Med. Press and Cir., August 18th.) A patient of his, a strong man working in an ice manufactory, suffered from it in an extreme degree—so much so that his fellow-workmen would not work by his side. The epidermis of the sole of the feet was white, as if macerated; there were small ulcerations in the furrows, and also around the nails. The odor was overpowering. Dr. Ortega prescribed baths of a solution of chloral (one in fifty) and wrapping the feet in a cloth dipped in a similar solution. Two days after the smell had entirely disappeared. Six days later, the treatment being continued, the ulcerations were less moist and covered with a layer of epidermis.—*Louisville Med. News.*

Sulphurous Acid.—The British Medical Journal reports the publication by Prof. Gamgee of a new and convenient mode of using sulphurous acid, the disinfecting qualities of which are universally known. Cold alcohol, the Professor asserts, will dissolve three hundred times its own volume of the gas, and a fluid possessing such powers of concentration can not but be as efficient as it is portable and convenient. A few drops of the sulphuretted alcohol in the bottom of a trunk will disinfect any clothing that may be put into it; and fungus germs, such as must in casks, etc., may be destroyed by the use of a very small quantity.

Sweating of Hands and Feet.—A correspondent sends us some leaves of the common plantain (*Plantago media,*) which he says are effectively used in his neighborhood for the cure of sweating of the feet. They are placed inside the shoes and are said to harden the skin and cure the excessive perspiration in a few days.—*Med. Press and Circular.*

On the Treatment of Jaundice with Large Doses of Ipecac.—Dr. Henry Cook, of Bombay, writes a suggestive article in *The Practitioner* on the above subject. Jaundice he divides, as usual into the hepatogenous and hæmatogenous. The more frequent cause of the former variety is, of course, a catarrh of the gall-ducts ; but in addition, there is often a form of hepatogenous jaundice, in India, at least, which comes on gradually, and is probably due to an obstruction or lesion of some kind in the minuter divisions of the hepatic duct. Hæmatogenous jaundice in India is oftenest due to malarial fevers. Now, in jaundice due to any of these three causes, but especially in the second form, he has found large doses of ipecac to act better than anything else. In one case he gave on the first day forty-five grains. Improvement began on the day following. On the third day he gave a dose of thirty grains, and two days later another dose of twenty grains. Improvement was steadily going on. In other cases two doses were sufficient to restore the normal action of the liver. Dr. Cook relates one case of hæmatogenous jaundice due to malarial fever, in which the large doses of ipecac apparently acted well.—*Medical Record.*

To Pass the Esophageal Tube sometimes is found very difficult, and dangerous delay may often be occasioned when the stomach-pump is required in cases of poisoning. In such cases the attempt is generally made to pass the tube with the patient in the dorsal position, and its passage is frequently obstructed at some point in the esophagus. This annoying difficulty usually may be overcome by holding the patient in the upright position during the passage of the tube. We do not know the author of this procedure, but remember having seen it successfully carried out in Bellevue Hospital, when all attempts to pass the tube, with the patient in the dorsal position, had failed.—*Chicago Medical Review.*

### THE ACTION OF BENZOATE OF SODA IN SCARLET FEVER AND DIPHTHERIA.

Dr. Demme makes the following statement in the yearly report of the Children's Hospital at Bern, published in the yearly report of the

*Allgemeine Wiener Medizinische Zeitung*, No. 24. He has treated twenty-seven cases of diphtheria with benzoate of soda internally and externally. Internally as large a dose as possible was given (five to twenty grams daily, dissolved in one hundred to one hundred and twenty-five grams of water, with the addition of one to one and a half grams of liquorice juice.) The external application was made by sprinkling the diphtheritic patches with alcoholic solution of benzoate of soda by means of an ordinary laryngeal insufflator. The applications were repeated every two to four hours.

If the local disease were spreading rapidly and the lymphatic glands of the throat were swollen, Dr. Demme injected benzoate of soda into the retromaxillary and submaxillary regions, and even into the swollen tonsils. Cold wrapping of the body was employed at the same time, for the lowering of the temperature, and cooling baths when there was severe fever. In septic forms of the disease he administered cognac (five to seventy-five grams daily.) Of the twenty-seven cases which were treated in this manner, six, or twenty-two per cent. died, which must be called very favorable when the severity of the case is considered. With regard to the special effect of benzoate of soda, Demme arrives at the following conclusions. 1st. Benzoate of soda is an effective antimycotic, both as an internal and an external application. 2d. The application of benzoate of soda, in the form of insufflation on the infected spot, favors the secretion of the mucous membrane and essentially promotes the separation of the diphtheritic deposit. 3d. A reduction of temperature is not produced by benzoate of soda. 4th. In all his cases Demme saw, under the continued use of benzoate of soda, an increase in intensity of the contractions of the heart, generally with diminution of their frequency, and increased discharge of urine. 5th. Benzoate of soda had no effect on nephritis, with regard to the secretion of albumen. The doses which produce a good effect in diptheria are, according to Demme, the following: for children from three to six months old, two and a half grams daily; from seven to twelve months, five grams; from one to two years, seven and a half grams; from three to seven years, twelve to fifteen grams daily. Demme never observed unpleasant symptoms after such doses.—*Medical and Surgical Reporter*.

### STIGMATA OF MAIZE.

Last winter, and again this spring, the *News* called the attention of its readers to corn-silk, technically stigmata of maize, as a remedy in nephritic and cystic troubles, etc. The medicinal properties of corn-silk were brought to the notice of the profession by Dr. Dufau, a French physician, in *Le Courrier Medical.* He commends the remedy in uric and phosphatic gravel, chronic cystitis, mucous and muco-purulent cystic catarrh, and in cardiac and nephritic dropsy. Dufau has given it without injury for three months at a time. He has known it to triple and even quintuple the quantity of urine passed in twenty-four hours. He says that in decoction it is unreliable and uncertain. He gives it in a syrup largely diluted, upon an empty stomach. Stigmata of maize is said to have been used time immemorial by the Mexicans.

Dr. Landrieux, of France, has published two cases showing its diuretic properties. The first was an individual with ascites from cirrhosis. Under the influence of the drug, given in a syrup, the urine arose rapidly from five hundred grams to twelve and fifteen hundred grams. In three weeks all ascites disappeared. The other case was the subject of heart-disease, with great edema of the legs, enormous ascites, pulmonary and renal congestion, and a considerable diminution of urinary excretion. The stigmata of maize increased the quantity of urine from two hundred to eight hundred grams in twenty-four hours. The edema and the ascites disappeared in a short time. Dr. Landrieux terminates his article thus: 1. Not only the different preparations of the stigmata of maize are useful as a modifying agent of the urine, but these same preparations can be equally considered as an incontestible diuretic agent; 2. Diuresis is rapidly produced; 3. The pulse becomes regular under its influence, the arterial tension increases, while that of the veins diminishes; 4. Complete tolerance of the drug, and in chronic cases the treatment might be continued during a month or six weeks without the slightest inconvenience.

We trust that some of our friends have tried this remedy and will write us the results. We have used it in a single instance, but with decided effect. Two double handfuls of corn-silk were boiled in two gallons of water until but a gallon remained. A tumblerful of this was given thrice daily to a patient of eighty, the subject of dropsy of the legs. His urine was scant, but a thorough examination failed to discover in the heart or kidney or liver any cause for the dropsy.

While taking the corn-silk decoction, which relieved his dropsy, he declared that he had never made so much water in all his life.

Professor Scheffer, of this city, is now preparing an extract of the stigmata of maize. Experiments must yet determine the time for gathering the silk, and the proper dose and best form of the remedy. It may be that the silk should be gathered before it is impregnated by the pollen from tassel.—*Louisville Medical News.*

Influence of Pilocarpine on Baldness.—The following occurs in the *Moniteur Scientifique* for February, 1880:

"Dr. G. Schmitz has twice noticed the reproduction of hair on the heads of bald patients, whom he had treated with hypodermic injections of pilocarpine for eye diseases (*Berl. Klin. Wochensch*). On an old man aged sixty, who had been operated on for double cataract, he had made three injections in the space of fourteen days. The membrane over the pupil disappeared, as he had expected, but at the same time the head of this man, who was completely bald, became covered with a thick down, and afterward his hair grew and became thicker, so that at the end of four months there was no trace of baldness left, and the patient became the possessor of an abundant crop of hair, partly white and partly black. In the case of another patient, thirty-four years old, suffering from detachment of the retina, the top of the head was entirely without hair on a surface as large as a playing-card. In this case, also, two injections of the same medicine resulted not only in curing the eye disease, but also in the reproduction of hair."

### IODOFORM SUPPOSITORIES FOR THE URETHRA AND CANAL OF THE CERVIX UTERI.

Editors Review:—For some months I have been experimenting with iodoform, trying to obtain some slowly soluble preparation which could be introduced into the urethra and the cervix uteri, so that the remedy would remain in direct contact with the diseased surface. The following is the formula I have obtained:

| | |
|---|---|
| Iodoform, | j oz. |
| Isinglass, | iv dr. |
| Gum Tragacanth (powd.), | j oz. |
| Glycerine, | ij dr. |
| Water (to dissolve ingredients), | i q. s. |

When sufficiently soft the mass is to be rolled into a cylinder small enough to be introduced into these canals and cut into pieces two inches in length. They can be introduced into the urethra of both male and female without the slightest difficulty. I introduced them into the canal of the cervix uteri in the following manner: I had made a tube, the bore of which is just large enough to admit the suppository, in which was fitted a loosely-working piston. A suppository is placed within the tube, and its mouth is carried up to the mouth of the cervix. With a light push on the handle of the piston the mass is made to enter the canal.

I have found iodoform used in this manner to be one of the most effective remedies in gonorrhœa and in diseases of the cervix uteri that I have ever resorted to. Yours truly,

C. N. FOWLER, M. D.

YOUNGSTOWN, O., September 10, 1880.

—*Ohio Med. and Surg. Review.*

### DENTISTS IN AMERICA.

It is not strange that twelve thousand dentists find employment in the United States. Dr. J. N. Farrar, of New York, states in an article published in the *Dental Laboratory*, that not less than half a ton of pure gold, costing about $500,000, is annually packed away in the mouths of Americans; and, in addition to this, there is probably four times as much cheaper material, such as silver, platina, etc., used in filling cavities in teeth. He makes the curious and interesting estimate that only three hundred years would be required to bury the amount of gold coin now in circulation in the country ($150,000,000) in the graveyards. The magnitude of American dental operations is shown by the statement that about three million artificial or porcelain teeth, mounted on various kinds of plates, are made every year. Dr. Farrar supplements these figures with the important statement, based on statistics compiled with painstaking labor, that out of an average of eighty people of all classes, only one can be found with perfect dental organs. The other seventy-nine require a dentist's care.

NITRITE OF AMYL IN UTERINE HEMORRHAGE.—Dr. Elias W. Kerr (British Medical Journal, November 1, 1879) quotes (Allg. Med. Central Zeitung, No. 1, 1879) Dr. Koehler as stating that, for the past seven years, in cases of uterine hemorrhage he has applied warm fomentations to the head to prevent anæmia of the brain, and also of the heart, and that by this means patients may be saved in the most dangerous cases of hemorrhage. Dr. Kerr says: "I can speak with confidence of the success of this treatment, and the rationale of it I take to be the rapid dilatation of the cerebral vessels; and if so, have we not in nitrite of amyl a more rapidly acting and more powerful agent to produce this result? The idea occurred to me a few nights ago, when attending a case of rather severe post-partum hemorrhage; and, happening to have some of the drug with me, I at once put my theory in practice by administering five minims by means of a Skinner's inhaler, and, I am happy to say, with immediate and most satisfactory result. The hemorrhage ceased at once and permanently, and the patient was restored from a state of collapse. Indeed, in this latter respect, its effect was only comparable to that of the hypodermic injection of ether. I have not been able to find any notice of nitrite of amyl having been heretofore used with the above views, and, of course, a single case is hardly sufficient datum upon which to claim much, but I hope to be able to give the medicine a more extended trial, and shall be glad to publish the results. *Chicago Med. Review.*

Cereus Bonplandii in Neuralgia.—R. E. Kunze, M. D. (*Therapeutic Gazette*), considers this remedy especially applicable in all conditions in which the sympathetic nervous system is involved to any extent. Cases of neuralgia of the heart, facial neuralgia, etc., have yielded to this remedy in a most satisfactory way.

Five drops every hour was the dose exhibited. A case of palpitation of the heart, of twenty-five years' standing, is reported cured by the same remedy in ten-drop doses four times a day.

### THE MEDICAL REGISTRATION LAW.

The law at present in force, regulating the licensing of practising physicians and surgeons in this city, is as comprehensive in its scope and as perfect in its detail as could be expected under the circumstances. Heretofore the mistake in all similar laws has been the aiming after impossibilities, and the consequence naturally has been that their provisions have been not only ignored, but the whole subject of medical legislation has been very justly brought into ridicule. The point of excellence in the law of this year is centred in the second section, which provides for a registration of all such as are or shall be legally qualified to practise medicine or surgery. Although this may have put many to an inconvenience, the latter is more than balanced by the good that must eventually accrue to each individual member of the profession. With such an end in view, the fact of compulsory registration is one with which the profession at large must be in accord. In fact, the willingness with which the great majority of medical men came forward to testify in a practical manner to the utility of the law, by complying with all its requirements, is a gratifying evidence of the truth of this assumption. From all parts of the State we hear that the legally qualified practitioners of medicine have been registering, and that the various county clerks have full lists of all medical men within their jurisdiction.

It is perhaps proper, in this connection, to call the attention of those physicians in this State, who may have neglected to register, to the fact that from the first of October they have rendered themselves liable to a fine of fifty dollars for the first offence of practising as an unregistered practitioner, and not less than one hundred dollars for each succeeding one. As one-half of such fines goes to the informer, it is easy to see that there may be peculiar inducements to some parties for making the necessary complaints. The provision of the law respecting the fines are so explicit that there does not seem to be a possibility of escaping a penalty when once its liability is proven.

There is no easier way of making a distinction between the legally qualified medical practitioner and the quack than the compulsory registration of qualifications. The qualifications fixed by law are so simple that none can misunderstand them, neither can any one complain that the standard of requirements is not within the reach of any ordinarily educated practitioner. It is this and other features of the new law which make it so consistent and so practicable. As there is now no legal excuse why even every professionally qualified medical man in this State should not be registered, there is still less to be said of such as are practising without license. To such medical men as believe that this law will be equally ineffective with previous ones, in suppressing irregular practices, we commend a careful reading of its provisions. It must be conceded that the framers of the bill understood the situation very thoroughly, and that every reasonable means have been used to prevent the illegal practice of medicine. The homœopaths and eclectics have equal rights with the regulars under the law, which is perfectly proper, and which will tend to divest the law of all supposed partisanship, and free it from all the elements of class legislation. Its whole aim is so clearly in the best interests o the people at large, that it can hardly fail to become as popular with outsiders as it already appears to be with the profession.

It remains to be seen what will be the effect of this law upon the irregular practitioners, men and women, who prescribe without any legal qualifications; clairvoyants, mesmerists, counter-prescribing druggists, and others of the same ilk. To continue their practices would be in open violation of the law, from the penalties of which there does not appear to be a reasonable hope of escape. It is explicitly stated that no one shall practise medicine or surgery in this State, without either having received a diploma or endorsement from a legally qualified medical college of this State, or from a Board of Examiners of the State University, evidence of either of which must be given by affidavit at the time of registering. As the registration is compulsory, hardly anything more can be desired to make the law effective. There are some features of the law which will have a tendency to reach beyond the mere suppression of quackery in some of its more pronounced forms. While much authority is vested in the medical colleges for granting diplomas, the State in its turn has a right to demand that the requirements for graduation shall come up to a certain standard. Failing to do this, such colleges can be deprived of their charter. By such means can colleges be prevented from disposing of their diplomas for shorter periods of study than required by law, and the bogus diploma trade can be suppressed.—*New York Medical Record.*

### THERAPEUTIC USES OF THE BROMIDES.

Rosenthal gives the following summary on this subject:

*Bromide of potassium* ought to be frequently suspended, because it produces loss of tone in the stomach, debility, and precordial pains. While it, in small doses, increases the appetite, in large doses it disturbs it, and consequently should be given largely diluted with milk.

*Bromide of sodium* is a preparation very salt and better tolerated by the stomach, and should be given in preference to old people, nervous people and children.

*Bromide of ammonium* is good in epilepsy and affections of the glottis, but is not superior to the other bromides.

*Bromide of camphor* moderates the action of the heart, and is good in alcoholism, in doses of fifteen to thirty grains.

*Bromide of zinc* cannot be given in pills, and is inferior to the other bromides.—*Virginia Medical Monthly.*

In New York, says the *Medical Record* of that city, the favorite bromide is, probably, bromide of sodium. The fact about the bromides is that one is just as good as another, the difference being in strength, not in quality. By varying the dose of bromide of potassium, for instance, nearly all the effects of the other bromides can be obtained.

In a mixture devised by Byasson, spermatozoa have been kept alive for twelve days, at a temperature of 36° C. The mixture is composed of water, 1,000 grammes, the white of one egg, and fifty-nine grammes of phosphate of soda. The liquid makes a useful injection in cases where sterility is due to excessive acidity of the utero-vaginal secretions.

At a recent meeting of the Adams County (Pa.) Medical Association, Dr. J. W. C. O'Neal, of Gettysburg, reported a prompt and efficient abortive treatment of boils and carbuncles, by the hypodermic injection of carbolic acid. The plan had been effectual in several cases of painful carbuncles, situated on the back of the neck, and in boils near the anus. Two of the medical gentlemen present, who had been subjected to the treatment, described the pain of the injection as being like the sharp sting of a wasp, lasting but three or four minutes, and then followed by complete relief, and subsequent resolution of the disease. Carbolic acid, pure, two-thirds to water one-third, was used, from six to eight drops of the solution being deeply injected into the centre of the tumor.

### TRACHEOTOMY SUPERSEDED.

In the *British Medical Journal*, July 24th, 31st, Dr. McEwen, of the Glasgow Royal Infirmary, advocates the use of tracheal tubes by the mouth instead of tracheotomy. He gives three cases in which he had recourse to the tubes, and their use was attended with very good results. Two were for the relief of œdema glottidis, and one to occlude hemorrhage from the larynx during an operation. The practical conclusions which he draws from these cases are as follows: 1. Tubes may be passed through the mouth into the trachea, not only in chronic, but also in acute affections, such as œdema glottidis. 2. They can be introduced without placing the patient under an anæsthetic. 3. The respirations can be perfectly carried on through them. 4. The expectoration can be expelled through them. 5. Deglutition can be carried on during the time the tube is in the trachea. 6. Though the patient at first suffers from a painful sensation, yet this passes off, and the parts soon become tolerant of the presence of the tube. 7. The patient can sleep with the tube *in situ*. 8. The tubes, in these cases at least, were harmless. 9. The ultimate results were rapid, complete and satisfactory. 10. Such tubes may be introduced in operations on the face and mouth, in order to keep blood from gaining access to the trachea, and for the purpose of administering the anæsthetic, and they answer this purpose admirably.

### DUTY OF PHYSICIANS.

We are not going to preach a sermon for physicians, says the *Canada Health Journal*, nor at them, but we believe there are but few in the profession who will not agree with us when we say, as we have before now repeatedly said in this journal, that a large part, and the noblest part, of the duty of medical practitioners lies in efforts to prevent disease. But we should be glad to see more practical manifestations of the exercise of this important duty.

If there are any of us in the profession who would say, "We are paid only, or almost only for *curing* disease, and often little enough for that. The public would not appreciate or thank us, much less pay us, for our efforts to *prevent* disease. There is often little enough work for us to do in our field of cure. Why should we exercise our skill in prevention?" let any such seek for an answer to this question in their own conscience—in their own hope of future happiness and peace of mind proceeding from well-doing.

But furthermore, to meet and satisfy the ever predominant instinct of self-preservation, it may be said that we believe it to be an invariable rule, that in always endeavoring to do what is best for our fellow-creatures we shall do what in the end will prove to have been best for ourselves.

The public might soon be taught, by united efforts on our part, to pay physicians for preventing disease. And the public could afford to pay much more liberally for prevention than for cure, while the practice of the profession would be easier and pleasanter, and its influence would become greater.

The following extract from the St. Louis *Courier of Medicine*, published by the Medical Journal Association of Missouri, has a ring about it with which we are much pleased:

"We have thought constantly, since the last meeting of the State Association, of how much we owe ourselves and our fellow-citizens and our successors, and how sadly deficient is our influence; and this ought not to be so. . . . The health of the people is the supreme law, and this should be, must be enforced. We (the profession) are in a condition to undertake the initiation of a movement that, if we commence in the right mode and unceasingly and unitedly endeavor to advance, will in time attain a force which I feel satisfied will overcome all the obstacles which now frown so threateningly in opposition—obstacles of custom, obstacles of ignorance, obstacles of prejudice, obstacles of intention. We must 'take up arms against this sea of troubles, and, by opposing, end them.' In this journal we purpose that one of the chief departments shall be devoted to matters relating directly and practically to public hygiene, urging the importance of the formation of local societies and boards of health, and this department will give constantly the latest results of the effect of attention to local causes of disease in this and foreign countries, and, in connection with this, instructive essays—simple, so plain and easy that he who runs may read and comprehend; often of so popular a character that the secular journals, the daily and weekly newspapers, shall copy them, and so commence the education of all people who read. We feel sure they will be read. Farmers and villagers are concerned in understanding drainage, the neglect of which has wrought sad disaster in our country. A ditch is cheap, and ditches are cut on every farm, but they need to be cut intelligently, and every country doctor ought to be able to tell his patients where one is needed, and where it had best lead. Wells are dug to form depots for drainage from cesspools, when a little needed knowledge would as easily avoid the lurking danger, that is only concealed by ignorance. Infected bedding and clothing have carried illness and death, by reason of expensive economy or death-dealing charity, because the infectiousness of filth or disease has been unknown, or the value of time as a purifier ignorantly estimated to be great. Oh, that we could vaccinate against wilful ignorance!"

### SELECTIONS.

APHORISMS ON INFANTILE MANAGEMENT AND MEDICATION.—From Dr. Amie M. Hale's little book on "The Management of Children:"

Of one thousand children born, one hundred and fifty die within twelve months. At fifteen years of age six hundred and eighty-four remain of the thousand.

The daily increase in weight of a normally-developing infant amounts to from a quarter of an ounce to three quarters of an ounce.

I consider bathing as the grand arcanum of supporting health, on which account, during infancy, it ought to be regarded as one of those sacred maternal duties, the performance of which should upon no account be neglected for a single day.

During the entire period of infancy and childhood the hair should be kept short. . . . I have never seen softer, better hair than on girls who have had it cut short, like that of school-boys, until they were in their tenth year.

Every article of dress worn during the day should be changed on retiring to rest.

The milk of the mother or of a healthy nurse is the natural and only proper food for an infant. Nature does not afford nor can art supply any substitute. In the asylums for foundlings and young infants, where feeding by hand has been substituted for the natural nourishment, the mortality has been most appalling. As high as ninety per cent. of the infants have been destroyed.

Never was there a more absurd or pernicious notion than that wine, ale or porter is necessary to a nursing-woman in order to keep up her strength, or to increase the quantity or to improve the nutritive qualities of her milk.

Children should not be allowed to eat frequently between meals. . . . The child should be accustomed to partake of food only at regular periods.

As a general rule, sugar should be given to children rather as an addition to less palatable articles of diet than as the principal food.

By a healthy child nearly all the saccharine fruits, when perfectly ripe and mellow, may be eaten in moderation with perfect safety.

